# Cost–benefit analysis of intervention reducing young stock mortality in Ethiopia

**DOI:** 10.3389/fvets.2024.1290705

**Published:** 2024-07-31

**Authors:** Thomas W. D. Kirk, Timothy Byrne, Paul Bessel, Ciara Vance, Christian Schnier, Andrew R. Peters

**Affiliations:** ^1^AbacusBio International Ltd., Roslin Innovation Centre, University of Edinburgh, Easter Bush Campus, Midlothian, United Kingdom; ^2^Epi Interventions Ltd., Tranent, United Kingdom; ^3^Supporting Evidence Based Interventions-Livestock, Royal (Dick) School of Veterinary Studies, University of Edinburgh, Easter Bush Campus, Midlothian, United Kingdom

**Keywords:** mortality, economics, smallholders, interventions, cattle, sheep, goats

## Abstract

Livestock provide meat, milk, draught labour, are used for breeding, and act as a store of value for smallholder farmers. High young stock mortality (YSM) has the potential to cause significant financial loss. The Young Stock Mortality Reduction Consortium collaborated on a project to deliver a package of basic health and husbandry interventions to reduce YSM for cattle and small ruminants in mixed and pastoral production systems in Ethiopia. Prior to the intervention, YSM rates ranged from 9.8% for calves in mixed systems, to 35.6% for small ruminants in pastoral systems. Proportional reductions YSM from the intervention ranged from 60% for calves and for small ruminants in mixed systems, to 72% for calves in pastoral systems. This brief research report assesses the costs and benefits of the intervention ex-poste to determine its efficiency. NPVs for the intervention (per household) were calculated for a range of benefit periods (from 1 to 20 years), based on the cost of training enumerators and farmers and the net annual household benefits realised within each benefit period. We found in both pastoral and mixed systems the net annual household benefit for the intervention was positive. For pastoral households the intervention achieves a positive NPV after 2 years. For mixed households the intervention achieves a positive NPV after 11 years. Overall, we found the benefits of the intervention exceed the costs, by a very large amount in pastoral systems, and that benefits were larger for households that kept larger numbers of breeding females.

## 1 Introduction

Ethiopia is home to 66 million cattle, 39 million sheep, and 47 million goats (1). Most livestock in the country are farmed in one of two major recognised production systems: mixed farming in the highlands, and pastoral farming in lowland areas (2). Livestock are an important asset for smallholder farmers because they provide meat and dairy output, draught labour, are used for breeding, and act as a store of value (3, 4). Interventions to improve the health and productivity of livestock have therefore been suggested as a strategy for poverty reduction and to improve the wellbeing of smallholder farmers and pastoralists (3, 5).

Mortality in young stock is one factor that affects the health and productivity of livestock (6, 7). Death of young animals reduces meat and dairy production, draught output, and also causes a loss of potential breeding females, which increases replacement costs, and makes it harder to expand the herd or flock in the future (6, 8).

Fentie (9) estimated mortality from birth to weaning for cattle in mixed systems, ranging from 9 to 14%, depending on the survey region. For cattle in pastoral systems, mortality ranged from 26 to 29% - similar to the results found in Ferede et al. (10). For small ruminants, Fentie (9) estimated mortality from birth to weaning ranged from 14 to 34% in pastoral systems, and 35 to 41% in mixed systems, again, depending on the region.

With the aim of reducing levels of mortality from birth to weaning, the Young Stock Mortality Reduction Consortium (YSMRC) was launched in 2016, led by the Ethiopian Government. Members of the YSMRC included Addis Ababa University’s College of Veterinary Medicine and Agriculture (CVMA-AAU) and Aklilu Lemma Institute of Pathobiology (ALIPB), the University of Gondar, the National Animal Health Diagnostic and Investigation Centre (NAHDIC), Tufts University, Supporting Evidence Based Interventions in Livestock (SEBI-Livestock), and The University of California, Davis.

The YSMRC collaborated on a pilot project to assess the impact of a package of basic health and husbandry interventions on calf, lamb, kid, and camel calf mortality in mixed, pastoral, and peri-urban production systems in Ethiopia. This brief research report presents results of the pilot study on bovine calf and young small ruminant (lamb and kid) mortality in pastoral and mixed systems only and assesses the costs and benefits of the intervention ex-poste to determine its efficiency. We define the costs and benefits of the intervention and quantify net present values (NPVs) of the intervention for a range of benefit periods, from 1 year up to 20 years after the intervention.

## 2 Methods

### 2.1 Study design and intervention

The pilot project involved delivering interventions to 900 households spread equally across six districts over five regions. These included two districts in regions where the main production system was pastoral (Awash Fentale in Afar, and Gursum in Oromia), and two districts in regions where mixed production was the main production system (Siyadabere & Wayou in Amhara, and Dalocha in the Southern Nations, Nationalities and Peoples region). The remaining 300 interventions were carried out in districts/regions where peri-urban production was the dominant system. We did not include peri-urban production systems in our analysis [previously described in Wong et al. (7)]. Within each of the six districts three kebeles/wards were selected (kebeles generally have three villages, each with around 150 households). Within each kebele, one village was purposively selected, with 50 households from that village then randomly selected, resulting in 150 households per district. We combined data from multiple districts to analyse pastoral and mixed systems overall.

Each intervention included a “minimum package of interventions.” Specific interventions varied somewhat between species and systems, and were designed to target the most important factors affecting young stock mortality in each system. Interventions were targeted at improving either farm management and animal husbandry practices, or directly improving animal health.

Examples of specific interventions aimed at improving management included preparing a clean area for newly born young stock and their mothers, improving final trimester diets for the dam, and providing preferential and separate calf feeding. Some specific interventions focused on prenatal care for the dam and neonatal care for the progeny. Other specific interventions aimed at prevention and control of diseases include deworming, and isolation and rehydration of young stock ill with diarrhoea. Comprehensive reviews of YSMRC intervention activities can be found in Wong et al. (7) and Allan et al. (11), which explore intervention impacts on epidemiological aspects of young stock mortality.

Baseline data for the trial was collected from March to August 2019, prior to the introduction of any of the interventions. The number of breeding females, the number of progeny born alive in the last 12 months, and the number of progeny born alive that died in the last 12 months were recorded per household. Young stock mortality was defined as the number of progeny born that died, divided by the number of progeny born alive, during the previous 12 months. The number of progeny born alive that survive to early maturity was calculated by subtracting the number of progeny that died from the number of progeny born (Table 1). This is equal to the number of progeny that were alive when the data was collected. Assuming animals are born uniformly throughout the year, the average age of these animals was 6 months, which is how we define “early maturity.” Additional data on the prevalence of respiratory diseases and diarrhoea was also collected, and is discussed in Wong et al. (7).

**Table 1:** Mean number of breeding females, mean number of progeny born alive during (the previous 12-month period), baseline and post-intervention mortality rates for young stock, baseline and post-intervention number of progeny surviving to 6 months, annual benefits (by species and total) from intervention (in ETB and USD), annual household cost and net annual household benefits, for cattle and small ruminants in pastoral and mixed production systems.

	Pastoral		Mixed	
	Cattle	Small ruminants	Cattle	Small ruminants
Breeding females per household^1^	10.0	29.6	2.1	4.1
Progeny born alive per household^1^	4.7	15.8	1.5	2.7
Mortality for young stock (%)				
*Baseline*	32.0%	28.0%	10.0%	40.0%
*Post-intervention*	9.0%	10.0%	4.0%	16.0%
% Reduction in mortality	−71.9%	−64.3%	−60.0%	−60.0%
Number of progeny surviving to 6 months				
*Baseline*	3.16	11.38	1.31	1.62
*Post-intervention^2^*	4.23	14.22	1.39	2.27
Change in number of progeny surviving to 6 months age	1.07	2.84	0.09	0.65
Annual benefit (by species)				
*Ethiopian Birr*	1,147	3,517	69	621
*US Dollar*	19.96	61.20	1.20	10.81
Annual household benefit (total)				
*Ethiopian Birr*	4,664		690	
*US Dollar*	81.16		12.01	
Annual household cost^3^				
*Ethiopian Birr*	1,799		280	
*US Dollar*	31.30		4.87	
Net annual household benefit				
*Ethiopian Birr*	2,865		410	
*US Dollar*	49.86		7.14	

^1^Mean calculated across multiple districts within production system (figures at baseline).^2^Calculated by applying the post-intervention mortality rate to the baseline number of progeny born.^3^Annual cost of materials is covered by the household receiving the intervention from year 2 onwards (the YSMRC covers material costs in year 1).

The interventions were introduced to participating farms via training sessions, where participating households were coached by extension officers throughout the 1-year study period.

Post-intervention evaluations were carried out from March to July 2020. The same categories of data were collected as for the baseline survey. The change in the number of progeny reaching 6 months of age was calculated by applying the post-intervention mortality rate to the number of progeny born alive during the 12 months prior to the intervention (Table 1), to control for differing numbers of progeny born alive during each 12-month period (as some natural fluctuation in the number of progeny born alive in each period would be expected).

### 2.2 Cost of the intervention

The total cost to deliver interventions to 900 households (including enumerator and farmer training, and intervention materials, and excluding survey monitoring costs) was 3.8 million Ethiopian Birr (ETB). This cost was equal to US $125,000 at the time the study was carried out (when 100 ETB = 3.3 USD), however, at June 2024 exchange rates (12) the total cost was equal to US $66,000 (when 100 ETB = 1.74 USD). For our analysis we focused on costs and benefits in ETB and have included US dollar equivalents for reference only.

The cost of training enumerators and providing farmer training was 3,192 ETB per household (for both mixed and pastoral households, or US $56). The intervention material costs (extra feed, disease treatments, etc.) was estimated at 45 ETB per breeding female per year (for both cattle and small ruminants), equal to 1,799 ETB and 280 ETB for pastoral and mixed households (US $31 and $5), respectively. The total intervention cost was paid for entirely by funders of the YSMRC in the first year only. We assume that material costs are incurred by households in subsequent years and there are no other recurring costs to households receiving the intervention.

### 2.3 Value of young animals

The direct impact of a reduction in young stock mortality is an increase in the number of progeny surviving to 6 months of age. We used existing sources for the value of young small ruminants. In the absence of market values for young cattle, to estimate the financial impact of the intervention we estimated the value of young cattle at 6 months of age.

We assume small ruminants reach maturity at 12 months of age. Because most YSM occurs prior to weaning, most small ruminants that reach 6 months of age will reach adulthood, therefore we used a base price of 900 ETB for adult sheep from Legese and Fadiga (13) for the value of small ruminants at 6-months of age in both pastoral and mixed systems. We multiplied the base price by a factor of 1.45 to account for inflation between 2014 and 2019 (the first year when intervention costs were incurred), based on the livestock component of the Ethiopian agricultural producer prices index (14), and calculated the value of a small ruminant (i.e., based on profit, rather than sale price) by adjusting for feed costs in pastoral and mixed systems (see below).

Cattle reach maturity much later than small ruminants, so it is not reasonable to value 6-month-old cattle based on adult cattle prices. Cattle generate value to farmers by providing food and income from meat and dairy output, draught labour, in breeding, and acting as a store of value (3, 4). Ultimately, the ability of an adult animal to provide all these outputs will be reflected in its sale price, which is in part determined by the animal’s weight (reflecting its ability to provide meat). A younger animal is not yet able to deliver some of these outputs (e.g., breeding or milk) and is only able to partially provide other outputs (e.g., meat); however, it still requires feed to be raised until it reaches fully productive adult age. The value of a young animal at any given time is therefore determined by the likelihood it survives to productive adult age (from its current age), its current weight (relative to its expected mature weight), the cost of feeding it until it reaches adulthood, and the price of an adult animal. This means the value of a young animal increases as it approaches adulthood because its weight and likelihood of survival to adult age both increase (survival increases as the time to adult age decreases). We calculated the value of an animal at a given age (*V_t_*) as follows:


$$V_{t}=P\times \frac{A}{a_{t}}\times \frac{w_{t}}{W}\times \left(1-f\right)$$

where *P* is the price of an adult animal, *A* is that proportion of animals born that reach adult age, *a* is the proportion of animals born that are still alive at time *t*, *w* is the animal’s weight at time *t,* W is the animal’s mature weight, and *f* is the lifetime feed costs as a proportion of the price of an adult animal.

For cattle, we used a southern system base price of 2,699 ETB and northern system cattle base price of 2,497 ETB from Gebre-Mariam et al. (2) for the price of an adult animal in pastoral and mixed systems, respectively. We multiplied each base price by a factor of 1.53 (14) to account for inflation between 2013 and 2019. We used baseline mortality rates (Table 1) to estimate the proportion of animals still alive at 6 months. We used annual offtake rates of 7 and 5% from Negassa and Jabbar (4) to estimate survival between ages 6 months and 2 years, in pastoral and mixed systems, respectively. Cattle typically reach a mature weight of 272 kg (15) at 2 years of age (assuming 50% carcase weight) and weigh 20 kg at birth (16). This implies an average daily growth rate of 0.34 kg per day, which was used to estimate the weight of cattle at time *t* (for both pastoral and mixed systems). Based on feed costs and cattle prices in Gebre-Mariam et al. (2) we estimated the feed costs as a proportion of sale price as 5% for pastoral systems and 27% for mixed. We used the same feed cost proportions for cattle and small ruminants.

**Figure 1:**
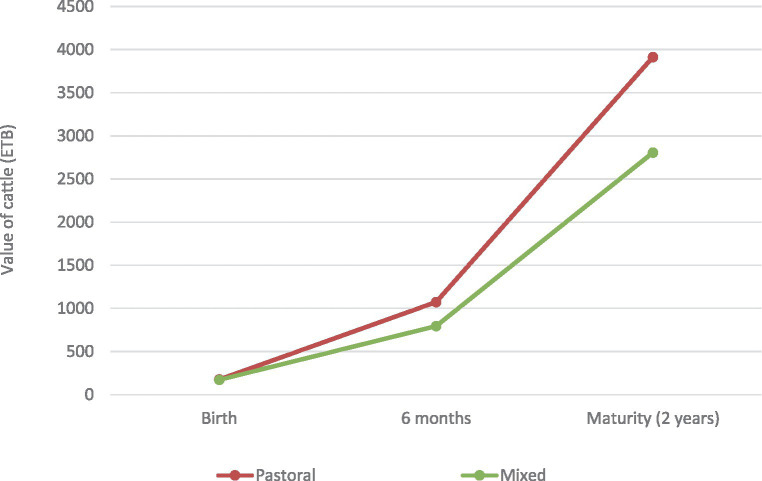
Estimated value of cattle, in pastoral and mixed production systems, at birth, 6 months, and 2 years of age, in Ethiopian Birr (ETB).

Using this approach, we calculated the value of young cattle at birth, 6 months, and 2 years of age (Figure 1).

### 2.4 Net present value of intervention over time

Annual household benefits for the intervention were calculated by multiplying the increase in the number of progeny surviving to 6 months by the value of an animal at age 6 months and combining cattle and small ruminant benefits. Material costs (paid for by the YSMRC in year 1, and by the household for subsequent years) were subtracted from the annual household benefits to calculate the net annual household benefit (Table 1). We also assume that the herd/flock size remains constant over time, meaning that additional surviving progeny are sold.

**Figure 2:**
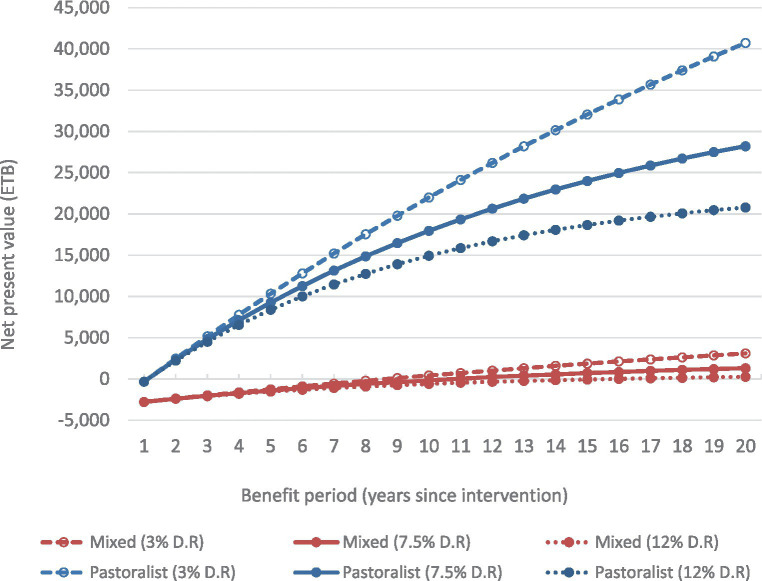
Net present value (using 7.5% discount rate) for range of benefit periods (from 1 year to 20 years after the intervention), for pastoral and mixed households, including sensitivity for lower (3%) and upper (12%) discount rate (D.R.) assumptions.

NPVs for the intervention for pastoral and mixed households were calculated for a range of benefit periods (from 1 to 20 years), based on the net annual household benefit and the initial cost of training enumerators and providing farmer training (incurred in year 1 only) and a 7.5% discount rate (Figure 2).

### 2.5 Sensitivity

Robinson et al. (17) suggests using a lower discount rate of 3% and an upper discount rate equal to twice the projected near-term GDP growth rate. Ethiopia is forecast to grow at nearly 6% in 2024 (18); therefore we used 12% as the upper discount rate. (Our main results use a discount rate equal to the average of the lower and upper rates.) We calculated sensitivity of the NPVs over time to the discount rate applied (Figure 2).

We also calculated sensitivity of the NPV to farm composition, that is, based on the combined number of cattle and small ruminant breeding females kept per household. Unlike the NPV results, which are calculated across range of benefit periods, sensitivity to farm composition is, by necessity, based on the costs and benefits within a specific benefit period. We calculated sensitivity of the NPV to farm composition after 10 years, which is the intermediate point of the NPV range (Table 2).

**Table 2:** Sensitivity of NPV after 10 years to the number of cattle and small ruminant breeding females kept per household, for pastoral and mixed systems.

Pastoral household^1^								
		Number of cattle (breeding females)						
		0	1	3	5	7	**10**	15
Number of female small ruminants	0	(3,192)	(2,681)	(1,659)	(637)	384	1,917	4,471
	1	(2,649)	(2,138)	(1,117)	(95)	927	2,460	5,014
	3	(1,564)	(1,053)	(31)	990	2,012	3,545	6,099
	6	64	575	1,596	2,618	3,640	5,173	7,727
	10	2,234	2,745	3,767	4,789	5,810	7,343	9,897
	20	7,660	8,171	9,193	10,215	11,237	12,769	15,324
	**30**	13,087	13,597	14,619	15,641	16,663	**18,195** ^**1**^	20,750
	40	18,513	19,024	20,045	21,067	22,089	23,621	26,176
Mixed household^1^								
		Number of cattle (breeding females)						
		0	1	2	5	10	15	20
Number of female small ruminants	0	(3,192)	(3,279)	(3,365)	(3,626)	(4,060)	(4,493)	(4,927)
	2	(1,628)	(1,715)	(1,801)	(2,062)	(2,496)	(2,929)	(3,363)
	**4**	(64)	(151)	**(237)** ^**1**^	(498)	(932)	(1,366)	(1,799)
	6	1,500	1,413	1,326	1,066	632	198	(235)
	8	3,064	2,977	2,890	2,630	2,196	1,762	1,328
	10	4,628	4,541	4,454	4,194	3,760	3,326	2,892
	15	8,538	8,451	8,364	8,104	7,670	7,236	6,802

^1^Where bold indicates a household with the average number of small ruminant and cattle breeding females (rounded to the nearest whole number).

## 3 Results

### 3.1 Value of young animals

The value of small ruminants at 6 months of age (in 2019) was calculated as 1,237 ETB and 958 ETB for pastoral and mixed systems, respectively. The value of cattle at age 6 months (in 2019) was calculated as 1,073 ETB for pastoral systems and 1,795 ETB in mixed systems (Figure 1).

### 3.2 Net present value of intervention over time

For pastoral and mixed systems, the intervention NPV is negative for a one-year benefit period (Figure 2), because the benefits from a single year are less than the upfront costs of training enumerators and providing farmer training. However, as the benefits period increases the intervention NPV increases, for both pastoral and mixed systems, because the annual household benefit is greater than the annual household cost. For pastoral households the intervention achieves a positive NPV within a two-year benefits period. After 10 years the NPV for pastoral households is 17,951 ETB (or US$312), which equates to a benefit–cost ratio of 2.09. For mixed households, it takes 11 years before the benefits of the intervention are greater than the costs. After 10 years the NPV for mixed households is −164 ETB (US $3), which equates to a benefit–cost ratio of 0.97; after 11 years the NPV is 35 ETB (US $0.6). There are diminishing marginal returns as the NPV period increases because benefits in the distant future are discounted by greater amounts. The difference between NPVs for pastoral and mixed households is due to differences in the number of animals kept by each. Larger herds/flocks translate to larger benefits for an otherwise equal decrease in mortality, therefore pastoral households receive larger benefits, while the cost of training enumerators and providing farmer training is the same for pastoral and mixed households.

### 3.3 Sensitivity

The sensitivity of the NPV to the annual discount rate increases over time. For example, the NPV after 5 years calculated using a 3% discount rate is only 14% higher than the NPV calculated using a 7.5% discount rate. This difference increases to 22% for NPV after 10 years, and 44% for the NPV after 20 years (Figure 2). Despite this, for pastoral systems, changing the discount rate does not affect the NPV being positive 1 year after the intervention. For mixed systems, using the lower discount rate results in the NPV becoming positive 2 years earlier (after 9 years) and using the upper discount rate results in the NPV taking 5 years longer to become positive (after 16 years). When assessing the appropriate discount rate to apply to smallholder farmer interventions, it should be noted that developing country respondents have shown strong time preferences (19). Strong time preferences imply that given the choice, respondents would prefer to receive less money now, than receiving more money in the future, which suggests higher discount rates are more appropriate than lower rates.

The 10-year NPV is sensitive to the number of cattle and small ruminant breeding females kept, where lower numbers are associated with a lower NPVs, and higher numbers are associated with larger NPVs (Table 2). For pastoral households the 10-year NPV can be positive even with relatively low numbers of cattle and small ruminant breeding females. [The 10-year NPV is very close to positive (−31, ETB, or US $1) for pastoral household with only three cows and three small ruminant breeding females.] In mixed systems the intervention does not have a positive NPV if the household keeps large numbers of cows only, but this is not true for small ruminants. For example, a mixed household with 20 cows has a negative 10-year NPV; however, a mixed household with only six small ruminant breeding females has a positive NPV (Table 2). This is because the annual benefit of the intervention for cattle in mixed systems is low, due to the very low baseline mortality in cattle (Table 1).

## 4 Discussion

The direct results from the intervention were a success, showing proportional reductions in mortality ranging from 60 to 72%. However, a cost–benefit analysis was essential for determining the ultimate outcome of the intervention, and how beneficial it was for the farmers receiving it.

Converting changes in mortality to increases in surviving progeny is important because gaining additional animals to provide milk, meat, draught labour etc. is how financial benefits manifest for farmers. It also revealed that the size of a herd/flock is critical in determining the ultimate impact of the intervention—a large reduction in mortality generates more surviving progeny, and more benefits, if there are larger number of animals being born in the first place. (This is why we found higher NPVs for pastoral systems than mixed systems.) Although the intervention does not have a positive 10-year NPV for households with few animals (including the average mixed household), due to the role livestock play in mitigating household shocks, for households whose only source of income is a few animals, the true benefits of avoiding mortality in these cases may exceed our market-based estimate, due to risks and dangers posed by complete loss of income. Further, the intervention itself may yield benefits over-and-above what is measured in this study. For example, better maternal health practices may improve production from breeding females, which may benefit all young stock (i.e., not only those at risk of mortality) in the form of better growth.

One issue raised is over what period is it appropriate to measure future benefits resulting from an intervention. Rather than defining an arbitrary benefits period we calculated NPVs for a range of benefit periods (from 1 to 20 years) and showed that the intervention for has a positive NPV after a very short time (2 years) for pastoral households. This was robust to the chosen discount rate. For mixed systems the intervention breaks even after 10 years but has greater NPV for households with above average numbers of small ruminant breeding females. Overall, the efficiency of the intervention is very good in pastoral systems, and good under certain conditions in mixed systems.

Given the small scale of the pilot project, if delivery was scaled up to reach a much larger number of households (e.g., by the state, or a private funder) it is plausible the cost per intervention could be reduced further (This would improve the efficiency of the intervention in mixed systems.) For example, the cost of training enumerators could be spread out over a larger number of intervention recipients. This raises the question if farmers would themselves be willing to pay to receive training. Positive NPVs from the intervention indicate the investment would pay for itself for farmers; however, the upfront cost may be a limiting factor.

We should also be aware that our results depend on an estimate for the value of young cattle. This is a limitation of this study. Placing an accurate financial value on livestock is critical for accurately estimating the benefits of any intervention. Future stakeholder surveys (for any livestock intervention assessment) should ask households receiving interventions at what price they would be prepared to sell their stock, thus providing a sensible estimate for the market value of any animals affected by an intervention.

## Data availability statement

Publicly available datasets were analysed in this study. This data can be found at: https://dataverse.harvard.edu/dataverse/livestock-lab-ethiopia-young-stock-mortality.

## Ethics statement

The animal studies were approved by Institutional Animal Care and Use committee at the University of California, Davis (Protocols # 19666 & 21,995) and the applicable ethics review committees at AAU-ALIPB and the University of Gondar. The studies were conducted in accordance with the local legislation and institutional requirements. Written informed consent was obtained from the owners for the participation of their animals in this study.

## Author contributions

TK: Conceptualization, Formal analysis, Investigation, Methodology, Writing – original draft, Writing – review & editing. TB: Writing – original draft. PB: Writing – original draft. CV: Conceptualization, Writing – original draft. CS: Methodology, Supervision, Writing – review & editing. AP: Funding acquisition, Writing – review & editing.
